# Tuning the Calcination Temperature of ZnO in Chitosan–Graphene Oxide/Epoxy Coatings for Optimized Corrosion Mitigation of Carbon Steel

**DOI:** 10.3390/polym18161959

**Published:** 2026-08-11

**Authors:** Yasin Albarqouni, Euodia Banius, Farah Alfoudari, Aljoury Alsulaiti, Mohammad R. Thalji, Arman Bin Abdullah

**Affiliations:** 1 Faculty of Chemical and Process Engineering Technology, Universiti Malaysia Pahang Al-Sultan Abdullah, Lebuhraya Persiaran Tun Khalil Yaakob, Kuantan 26300, Pahang, Malaysia; euodiabanius@gmail.com; 2 Faculty of Applied Medical Sciences, Clinical Nutrition Department, Al–Azhar University-Gaza, Gaza City 1277, Palestine; 3 School of Sustainable Engineering and the Built Environment, Arizona State University, Tempe, AZ 85287, USA; frsfoudari@gmail.com (F.A.); jnssulaiti@gmail.com (A.A.); 4 Korea Institute of Energy Technology (KENTECH), 21 KENTECH-gil, Naju 58330, Jeollanam-do, Republic of Korea

**Keywords:** chitosan–graphene oxide, zinc oxide, calcination, corrosion protection, epoxy coating, electrochemical impedance spectroscopy

## Abstract

The corrosion-protection performance of zinc oxide-hybrid polymeric coatings is traditionally attributed to the individual contributions of their constituent phases. This study reveals that the calcination temperature of zinc oxide (ZnO) filler is a critical, previously overlooked processing parameter that dictates not only filler crystallinity but also the collective synergistic failure mechanism of the entire coating system. Herein, we demonstrate that incorporating ZnO calcined at 500 °C yields a ternary chitosan–graphene oxide–zinc oxide/epoxy (CS–GO–ZnO/EP) composite coating with a highly compact, dense morphology, minimal internal porosity, and exceptional filler dispersion, as validated by FTIR, XRD, and SEM analyses. The optimized CS–GO–ZnO/EP coating applied to carbon steel exhibits outstanding dry and wet pull-off adhesion strengths, the highest surface hydrophobicity (102.2°), and superior electrochemical barrier protection. Notably, after a 120-h immersion period in an aggressive 3.5 wt.% NaCl electrolyte, the CS–GO–ZnO/EP (500 °C) maintains excellent coating resistance (R_coat_ = 1.06 × 10^5^ Ω) and a minimized corrosion rate (CR = 0.074 mm/y). This thermal threshold is a key processing window that improves chemical bonding and compatibility between the different parts of the hybrid matrix without causing the severe nanoparticle sintering, phase aggregation, and micro-cracking that happen at 650 °C. This work offers a significant advancement in the design of eco-friendly, high-performance hybrid coatings, demonstrating that precise control of the inorganic phase’s thermal history provides a direct pathway toward superior durability, hydrophobicity, and electrochemical stability for carbon steel protection in aggressive marine environments.

## 1. Introduction

Carbon steel is one of the most extensively used engineering materials owing to its excellent mechanical properties, low cost, and ease of fabrication. However, its high susceptibility to corrosion in aggressive environments containing moisture, dissolved oxygen, carbon dioxide, hydrogen sulfide, and chloride ions significantly limits its long-term durability and reliability. Corrosion-induced degradation not only compromises the mechanical integrity of steel structures but also poses safety risks, equipment failures, and substantial economic losses [1,2]. It has been estimated that corrosion accounts for approximately 2–4% of the gross domestic product of many countries, representing nearly one-third of global industrial production losses associated with metallic materials. Consequently, the development of efficient and environmentally sustainable corrosion-protection technologies remains an important scientific and industrial challenge.

Among the available corrosion mitigation strategies, polymeric coatings have emerged as one of the most practical and cost-effective approaches because they provide a physical barrier that isolates metallic substrates from corrosive species. Epoxy-based coatings, in particular, have attracted widespread attention owing to their excellent adhesion, chemical resistance, and mechanical properties. Nevertheless, conventional epoxy systems suffer from limitations associated with environmental concerns, brittleness, and susceptibility to microcrack formation during long-term service, which can compromise their protective performance [3]. Therefore, considerable research efforts have focused on developing environmentally friendly polymer-based composite coatings that combine superior barrier properties with enhanced mechanical stability and long-term corrosion resistance [1,2,4,5]. Recent advances in polymer nanocomposite coatings have shown that incorporating functional nanostructures can significantly enhance environmental stability and corrosion resistance through synergistic interfacial interactions. For example, copper nanowires (Cu NWs) encapsulated with conducting polypyrrole (PPy) have been reported to exhibit excellent oxidation resistance, long-term stability, and enhanced protection against environmental degradation owing to the formation of a uniform polymer shell around the metallic nanowires [6]. Similarly, ultrathin PPy-coated Cu NWs have shown remarkable environmental stability while preserving their intrinsic functional properties, highlighting the effectiveness of conductive polymer coatings in protecting reactive metallic components from oxidation and corrosion [7]. These studies demonstrate the importance of nanofiller engineering and polymer–nanomaterial interfacial design in developing advanced, high-performance protective coatings.

Chitosan (CS), a naturally derived polysaccharide, has emerged as a promising sustainable polymer for protective coatings because of its biodegradability, non-toxicity, excellent film-forming ability, metal-chelating capability, and strong adhesion to metallic substrates [8,9]. Compared to conventional synthetic polymers, CS offers distinct advantages for protective coating applications: renewable sourcing from biomass, processing in aqueous acidic media without toxic organic solvents, intrinsic metal-chelating capability, and abundant –NH_2_ and –OH groups that enhance strong chemisorption onto steel surfaces [10]. These features make chitosan an attractive eco-friendly alternative to petroleum-based polymers, while incorporating GO and ZnO nanofillers effectively overcomes its mechanical limitations. Despite these advantages, pure CS coatings generally exhibit poor mechanical strength, limited stability in aqueous or marine environments, and insufficient long-term durability, which restrict their practical applications under harsh service conditions [11]. Consequently, reinforcing CS with suitable inorganic nanofillers has become an effective strategy for improving the structural integrity and protective performance of polymer coatings. Among various nanofillers, graphene oxide (GO) has attracted considerable interest because of its two-dimensional (2D) layered structure, high aspect ratio, and abundant oxygen-containing functional groups. These characteristics not only create highly tortuous diffusion pathways that effectively retard the penetration of water, oxygen, and chloride ions but also promote excellent dispersion and strong interfacial interactions within polymer matrices through hydrogen bonding and other intermolecular forces [12]. Zinc oxide (ZnO), an environmentally benign n-type semiconductor, has also been widely incorporated into polymer coatings owing to its excellent chemical stability, ultraviolet shielding, antimicrobial activity, and corrosion-inhibiting properties [13]. When homogeneously dispersed within a polymer matrix, ZnO can enhance coating compactness, improve mechanical strength, and reduce defect density, thereby increasing the barrier efficiency against corrosive media. The performance of ZnO is strongly influenced by its physicochemical characteristics, including crystallinity, particle size, morphology, surface defects, and specific surface area, all of which are governed by the calcination process [14]. Increasing the calcination temperature generally enhances crystallinity and phase stability by eliminating residual organic species and structural defects. However, excessive calcination may promote particle growth and agglomeration, leading to reduced surface area and weaker interactions with the surrounding polymer matrix [15]. Such thermally induced structural changes are expected to influence nanoparticle dispersion, polymer–nanofiller interfacial compatibility, coating compactness, and ultimately the corrosion-protection performance of polymer nanocomposite coatings. Nevertheless, these relationships remain insufficiently understood.

While binary composite epoxy coatings incorporating CS–ZnO/EP, CS–GO/EP, or GO–ZnO/EP have demonstrated improved corrosion protection compared to pristine epoxy or individual filler systems, these two-component configurations still suffer from inherent limitations. CS–ZnO binary systems exhibited inadequate barrier properties owing to the absence of high-aspect-ratio 2D nanosheets that could effectively extend the diffusion pathways for corrosive species [16]. Similarly, CS–GO binary composites lacked the active corrosion-inhibiting functionality of ZnO, which releases Zn^2+^ ions to form passive protective films at cathodic sites. GO–ZnO binary systems, while offering improved barrier and inhibition properties, suffer from poor interfacial compatibility and limited adhesion to metallic substrates due to the absence of CS’s abundant polar functional groups, including amide and hydroxyl groups, that facilitate strong chemisorption onto steel surfaces.

The design of a ternary CS–GO–ZnO hybrid system overcomes these individual shortcomings through a synergistic combination of three complementary functionalities. CS serves as a macromolecular bridging agent that enhances interfacial adhesion and promotes uniform filler dispersion; GO provides a highly tortuous physical barrier network that impedes electrolyte penetration, and ZnO acts as an active corrosion inhibitor that releases Zn^2+^ ions to passivate the underlying steel substrate. This three-component synergy is expected to yield superior corrosion protection performance that would not be achieved by any binary combination alone. However, the effectiveness of this ternary system is critically dependent on the physicochemical properties of ZnO, which are governed by its thermal processing history. Therefore, this study aims to investigate the influence of ZnO calcination temperature on the structural, morphological, and electrochemical properties of ternary CS–GO–ZnO/epoxy hybrid coatings deposited on carbon steel. By establishing the relationship between ZnO thermal treatment, polymer-nanofiller interactions, coating microstructure, and corrosion resistance, this work aims to identify the optimal calcination condition that maximizes the synergistic protective performance of the ternary system for carbon steel protection in aggressive chloride-rich environments.

## 2. Materials and Methods

### 2.1. Materials

Zinc acetate dihydrate (Zn(CH_3_CO_2_)_2_·2H_2_O), oxalic acid (C_2_H_2_O_4_·2H_2_O), acetic acid (CH_3_COOH), and sodium chloride (NaCl) were purchased from R&M Chemicals Co., Ltd. (Kuala Lumpur, Malaysia). Chitosan was supplied by Chemiz (M) Sdn. Bhd. (Selangor, Malaysia), while graphene oxide was obtained from XFNANO, Nanjing, China. Absolute ethanol (EtOH), provided by the HmbG (Hamburg, Germany), was used as the solvent throughout the synthesis process. A commercial epoxy system consisting of Araldite epoxy resin (Huntsman) and HY-951 aliphatic amine hardener was purchased from Huntsman Co., Bergkamen, Germany. Carbon steel coupons (1 cm × 5 cm × 0.1 cm) were used for electrochemical measurements, whereas larger coupons (5 cm × 5 cm × 0.1 cm) were employed for adhesion testing. All chemicals were of analytical grade and used as received without further purification.

### 2.2. Synthesis of ZnO Nanoparticles

ZnO nanoparticles were synthesized via a sol–gel method following the procedure reported previously [17]. Briefly, 11 g of Zn(CH_3_CO_2_)_2_·2H_2_O was dissolved in 100 mL of EtOH and refluxed at 60 °C for 30 min. Separately, 17.65 g of C_2_H_2_O_4_·2H_2_O was dissolved in 200 mL of EtOH. The oxalic acid solution was slowly added to the zinc acetate solution under continuous stirring, and then the mixture was refluxed at 50 °C for 1 h. The resulting precipitate was filtered, washed thoroughly with EtOH, and dried at 80 °C for 12 h. The dried precursor was ground into a fine powder using an agate mortar and divided into three equal portions. One portion was retained without calcination, while the remaining portions were calcined in a muffle furnace at 500 °C and 650 °C for 4 h using a heating rate of 5 °C min^−1^.

### 2.3. Preparation of CS–GO–ZnO Composite

Graphene oxide (0.6 g) was dispersed in 150 mL of distilled H_2_O under mechanical mixing for 30 min. Separately, CS (2.0 g) was dissolved in EtOH containing 1% (*v*/*v*) CH_3_COOH and stirred for 3 h to obtain a homogeneous gel. The GO suspension was subsequently mixed with the CS gel under continuous stirring. ZnO nanoparticles (0.6 g) obtained from each calcination condition (uncalcined, 500 °C, and 650 °C) were dispersed in 20 mL of EtOH and gradually incorporated into the CS–GO mixture until a homogeneous paste was formed. The resulting composite was dried at 150 °C for 8 h and subsequently ground into a fine powder for further use [18].

### 2.4. Structural and Morphological Characterization

The chemical structure of the synthesized ZnO nanoparticles and CS–GO–ZnO composites was analyzed using Fourier transform infrared spectroscopy (FTIR, Spectrum 100, PerkinElmer, Waltham, MA, USA). Crystalline phases were identified by X-ray diffraction (XRD, Miniflex II, Rigaku Corporation, Tokyo, Japan) employing Cu-Kα radiation (λ = 1.5406 Å) operated at 30 kV and 15 mA over a 2θ range of 10–80°. Surface morphology and microstructural characteristics were examined using field-emission scanning electron microscopy (FE-SEM, JSM–7800F, JEOL Ltd., Tokyo, Japan).

### 2.5. Coating Preparation and Surface Characterization

Epoxy coatings were prepared by mixing Araldite epoxy resin with HY-951 hardener at a weight ratio of 2:1. Each coating formulation contained 2 wt.% additive, corresponding to the optimum loading reported previously [19]. Accordingly, 0.1 g of ZnO, GO, or the CS–GO–ZnO composite was dispersed in 4.9 g of the epoxy system and mixed thoroughly to ensure uniform dispersion. Before coating, carbon steel substrates were sequentially polished using silicon carbide abrasive papers of increasing grit size, rinsed with ethanol, and dried. The coatings were applied with a doctor blade to achieve a uniform thickness of approximately 100 μm, as verified with a coating thickness gauge. All coated specimens were cured at room temperature for 120 h before characterization. Adhesion strength was evaluated in accordance with ASTM D4541 using a pull-off adhesion tester. Surface wettability was determined by static water contact angle measurements using a contact angle analyzer (LAUDA OSA100, LAUDA Scientific GmbH, Lauda-Königshofen, Germany). The surface topography of the coated specimens was further characterized by atomic force microscopy (AFM, Park NX10, Park Systems, Suwon, Republic of Korea). Contact-mode measurements were performed over a scanning area of 30 × 30 μm^2^ using a resolution of 256 pixels per line and a scan rate of 0.60 Hz. Multiple regions were analyzed for each specimen to ensure representative surface characterization. The obtained images were processed using Gwyddion software (Version 2.67). Quantitative surface roughness parameters, including root mean square roughness (Sq), mean roughness (Sa), and surface slope (Sdq), were extracted from the AFM images using the statistical analysis module of the Gwyddion software. At least three different scan areas per specimen were analyzed, and the results are reported as mean ± standard deviation.

### 2.6. Electrochemical Measurements

Electrochemical performance was evaluated using a Metrohm Autolab PGSTAT302N potentiostat workstation in a conventional three-electrode cell containing 3.5 wt.% NaCl electrolyte at room temperature. Before testing, the coated carbon steel specimens were fully cured for 120 h. A coated carbon steel coupon with an exposed area of 2 cm^2^ served as the working electrode. In contrast, an Ag/AgCl electrode and a platinum wire were used as the reference and counter electrodes, respectively. Open-circuit potential (OCP) measurements were first conducted for 3600 s to achieve a stable electrochemical state. Electrochemical impedance spectroscopy (EIS) measurements were subsequently performed by applying a 5 mV sinusoidal perturbation over a frequency range of 100 kHz to 0.01 Hz. Potentiodynamic polarization (PDP) measurements were carried out by scanning the potential from −0.5 to +1.0 V relative to the OCP at a scan rate of 1 mV s^−1^. All electrochemical measurements were performed in triplicate using independently prepared coating samples to ensure reproducibility. The experimental data were analyzed using NOVA 1.1 and OriginPro 2016 software. Corrosion parameters, including corrosion potential (E_corr_), corrosion current density (I_corr_), anodic Tafel slope (βa), cathodic Tafel slope (βc), polarization resistance (Rp), and corrosion rate (CR), were determined by Tafel extrapolation of the PDP curves. The EIS spectra were fitted using an equivalent electrical circuit comprising the solution resistance (R_s_), coating resistance (R_coat_), coating constant phase element (CPE_coat_), charge-transfer resistance (R_ct_), and double-layer constant phase element (CPE_dl_).

### 2.7. Statistical Analysis

All experiments were performed in triplicate using independently prepared samples to ensure reproducibility. The experimental results are presented as the mean ± standard deviation (SD). Water contact angle and electrochemical measurements were repeated using independently prepared specimens under identical experimental conditions. Electrochemical impedance spectroscopy (EIS) data were fitted using NOVA 1.1 software, while data processing and graphical analyses were performed using OriginPro 2016. The quality of the equivalent circuit fitting was evaluated using a chi-square (χ^2^) goodness-of-fit criterion, and values below 10^−3^ were considered indicative of acceptable fitting accuracy.

## 3. Results and Discussion

### 3.1. Structural Characterization

**Figure 1:**
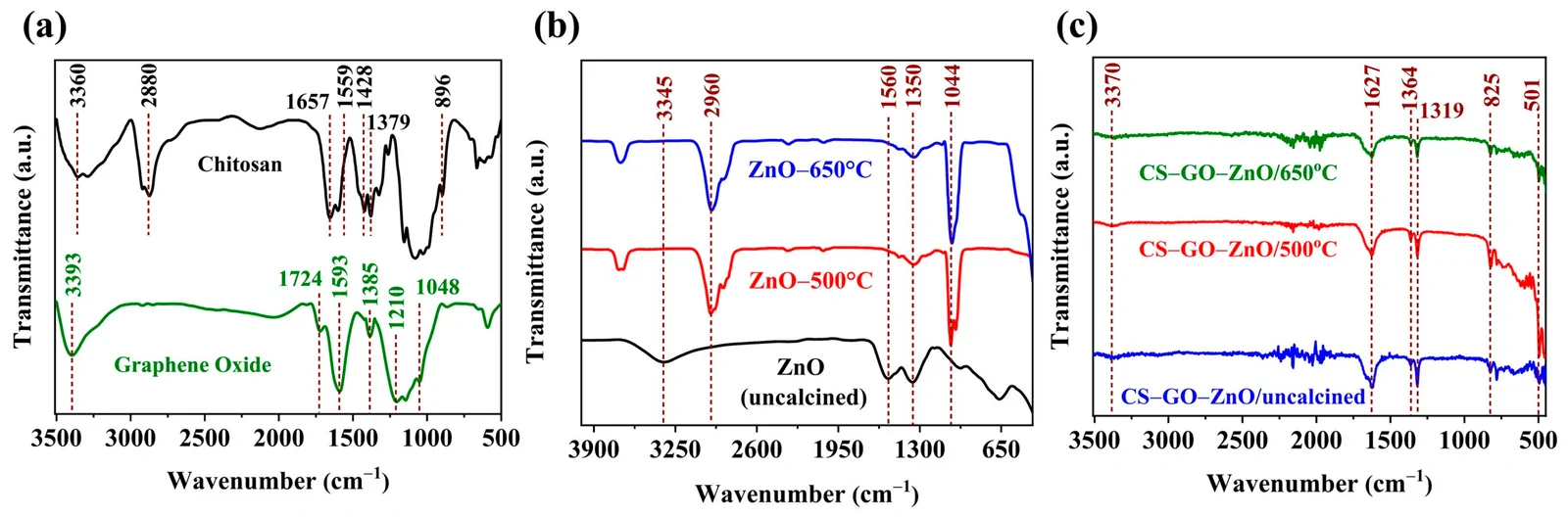
Fourier transform infrared (FTIR) spectra of (**a**) chitosan (CS) and graphene oxide (GO), (**b**) ZnO nanoparticles synthesized without calcination and after calcination at 500 and 650 °C, and (**c**) CS–GO–ZnO hybrid composites prepared using ZnO nanoparticles calcined at different temperatures.

The FTIR spectra of CS, GO, and ZnO prepared under different calcination temperatures, and the corresponding CS–GO–ZnO composites, are presented in Figure 1. As shown in Figure 1a, CS exhibited a broad absorption band at 3360 cm^−1^, corresponding to the overlapping stretching vibrations of –OH and N–H groups. The absorption band near 2880 cm^−1^ is assigned to C–H stretching vibrations, while the peaks located at 1657 and 1559 cm^−1^ are attributed to the amide I (C=O stretching) and amide II (N–H bending) vibrations, respectively. Additional characteristic bands at 1379 and 896 cm^−1^ correspond to C–H bending and β-glycosidic linkages of the chitosan backbone. In contrast, GO exhibited a broad O–H stretching band at approximately 3393 cm^−1^, together with characteristic peaks at 1724 cm^−1^ (C=O stretching of carboxyl groups), 1593 cm^−1^ (aromatic C=C skeletal vibration), 1385 cm^−1^ (C–OH deformation), 1218 cm^−1^ (epoxy C–O–C stretching), and 1048 cm^−1^ (alkoxy C–O stretching) [20], confirming the presence of abundant oxygen-containing functional groups on the GO surface [21,22]. The FTIR spectra of ZnO nanoparticles synthesized under different thermal treatments (uncalcined, 500 °C, and 650 °C) are presented in Figure 1b. The uncalcined ZnO exhibited a broad hydroxyl absorption band associated with adsorbed water and surface hydroxyl groups. Following calcination at 500 and 650 °C, this band shifts toward higher wavenumbers (approximately 3345–3370 cm^−1^), indicating the progressive removal of physically adsorbed water and surface hydroxyl species, consistent with previous reports [23]. A characteristic absorption band centered at approximately 2960 cm^−1^ appears after calcination and has been attributed to Zn–C–H stretching vibrations associated with residual surface carbon species [24]. Importantly, an absorption band at 1044 cm^−1^ emerged in calcined ZnO samples. This band is attributed to Zn–O vibrations associated with surface hydroxyl (Zn–OH) species and became more pronounced after calcination, suggesting improved structural ordering and crystallinity of the ZnO nanoparticles following thermal treatment [25]. Furthermore, the disappearance of the carbonyl (C=O) absorption originating from residual oxalate precursors demonstrated the effective decomposition of organic species during thermal treatment. The absorption band observed near 500 cm^−1^ corresponds to Zn–O lattice vibrations, confirming the successful formation of crystalline ZnO nanoparticles [17]. These results indicate that calcination effectively removes residual impurities while improving the structural purity of ZnO, thereby providing cleaner nanoparticle surfaces for interaction with the polymer matrix.

The FTIR spectra of the CS–GO–ZnO composites prepared using ZnO nanoparticles calcined at different temperatures are shown in Figure 1c. All composites exhibited a broad absorption band centered at 3370 cm^−1^, corresponding to hydrogen-bonded –OH and –NH stretching vibrations derived from CS and GO components. The characteristic bands observed at 1627, 1364, and 1319 cm^−1^ are assigned to amide I/C=C stretching, C–H deformation, and C–N stretching vibrations of the CS framework, respectively. In contrast, the absorption band near 825 cm^−1^ is associated with Zn–OH vibrations, confirming the successful incorporation of ZnO nanoparticles into the CS–GO matrix [26]. In addition, the Zn–O stretching vibration remains evident at 501 cm^−1^, demonstrating that the crystalline ZnO structure is preserved after composite fabrication. Compared with the pristine components, slight shifts in both band positions and intensities are observed after composite formation, suggesting the establishment of strong hydrogen bonding and interfacial interactions between the hydroxyl and amino groups of CS, the oxygen-containing functional groups of GO, and the ZnO nanoparticle surfaces. These interactions indicate the successful formation of an integrated CS–GO–ZnO hybrid network, which is expected to enhance the structural integrity and barrier performance of the subsequent epoxy coating.

**Figure 2:**
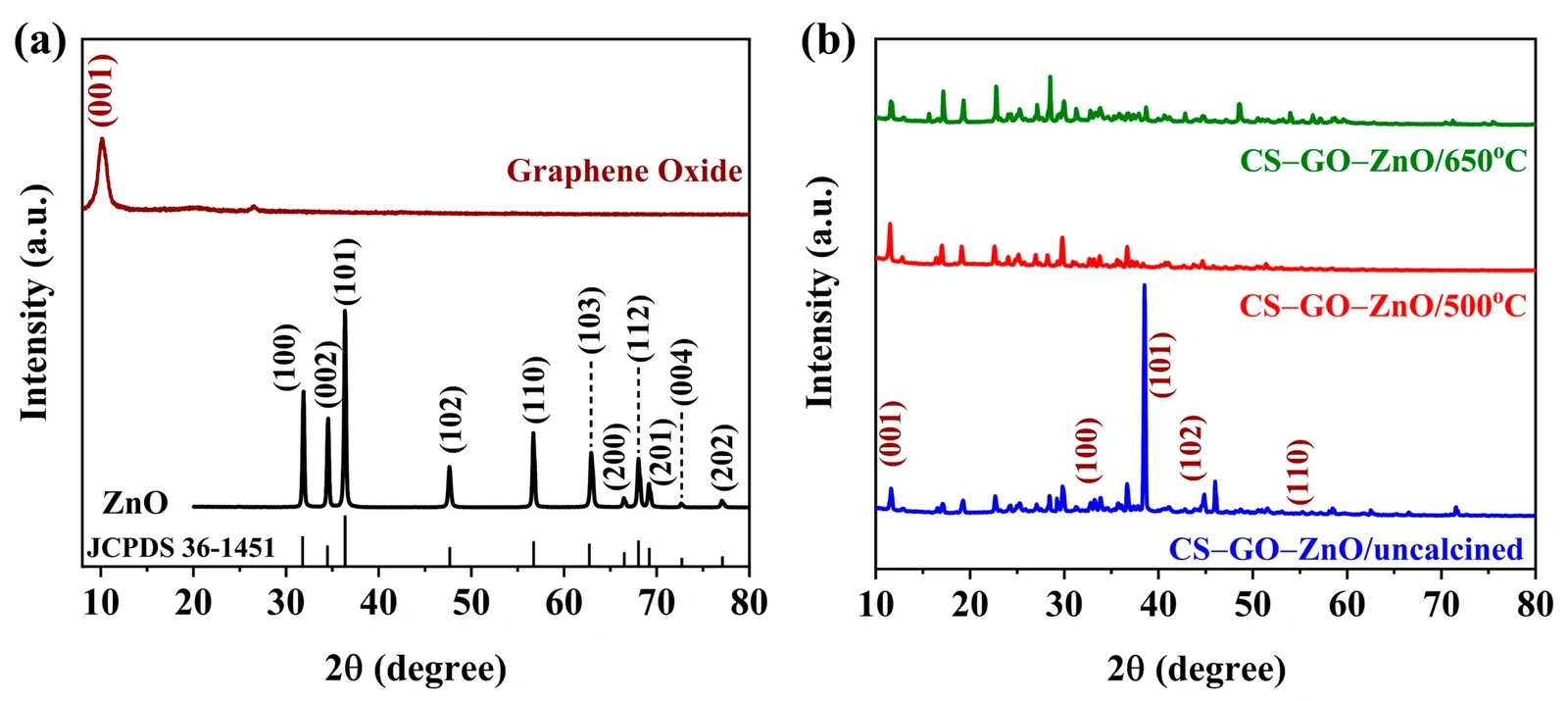
XRD patterns of (**a**) graphene oxide and hexagonal ZnO and (**b**) CS–GO–ZnO composites prepared using ZnO nanoparticles calcined at different temperatures.

The XRD patterns of GO, the reference hexagonal ZnO phase (JCPDS No. 36-1451), and the CS–GO–ZnO hybrid composites prepared using ZnO nanoparticles synthesized under different calcination temperatures are presented in Figure 2. As shown in Figure 2a, GO exhibited a characteristic diffraction peak at 2θ = 10.1°, indexed to the (001) plane, confirming the presence of oxygen-containing functional groups that increase the interlayer spacing of graphene sheets. The reference diffraction positions of hexagonal wurtzite ZnO (JCPDS No. 36-1451) are also included for comparison, with the principal reflections corresponding to the (100), (002), (101), (102), (110), (103), (200), (112), (201), (004), and (202) planes at 2θ of 31.9°, 34.5°, 36.3°, 47.7°, 56.7°, 62.9°, 66.5°, 68.1°, 69.2°, 72.7°, and 77.1°, respectively. The excellent agreement between these standard reflections and the diffraction peaks observed in the composite patterns confirms that the ZnO nanoparticles retained their hexagonal wurtzite crystal structure after synthesis and subsequent composite fabrication.

The XRD patterns of the CS–GO–ZnO composite fillers prepared at varying calcination temperatures are shown in Figure 2b. All samples exhibited the characteristic GO (001) reflection at approximately 11.4°, indicating that the layered GO structure is preserved after incorporation into the hybrid composite. In addition, diffraction peaks corresponding to the (100), (101), (102), and (110) planes at 2θ of 33.3°, 38.5°, 44.7°, and 54.6° of hexagonal ZnO remained clearly visible, confirming the successful incorporation of crystalline ZnO into the CS–GO matrix. No additional diffraction peaks associated with secondary crystalline phases were detected, indicating that the composite fabrication process did not alter the crystal structure of ZnO. Compared with the uncalcined sample, the composites prepared using ZnO calcined at 500 and 650 °C exhibited a more uniform distribution of diffraction intensities and a slight reduction in the dominant (101) peak intensity. These changes suggest that thermal treatment modifies the crystallinity and crystallite growth of ZnO before incorporation into the polymer matrix while maintaining its crystal phase. The preservation of the characteristic ZnO reflections together with the GO diffraction peak demonstrates the successful integration of ZnO nanoparticles within the CS–GO hybrid network. Furthermore, the absence of impurity peaks and the retention of the characteristic diffraction features indicate good structural compatibility among the three constituents, providing a stable framework for the subsequent fabrication of epoxy-based composite coatings [19].

**Figure 3:**
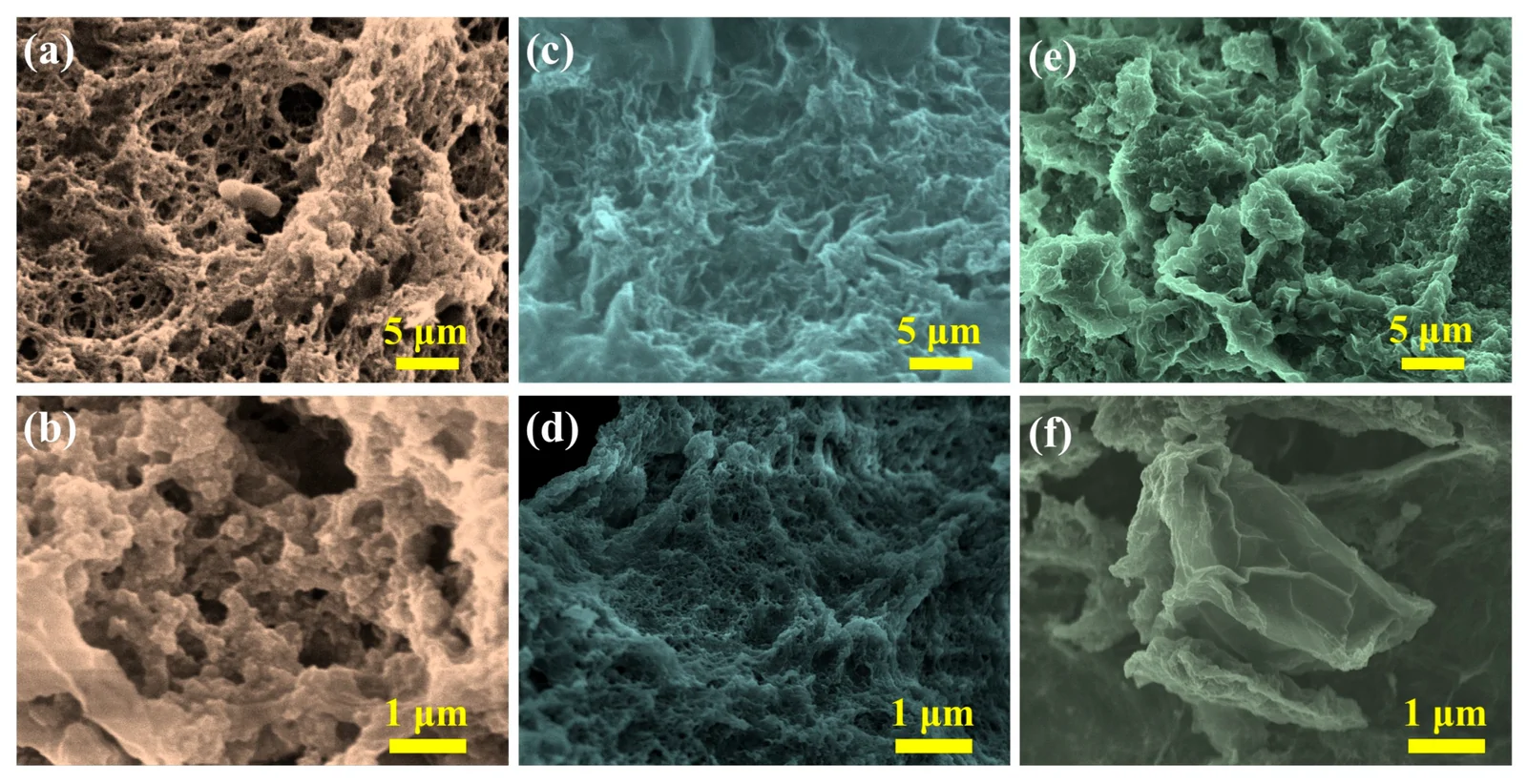
FE-SEM images of CS–GO–ZnO composites prepared using (**a**,**b**) uncalcined ZnO, (**c**,**d**) ZnO calcined at 500 °C, and (**e**,**f**) ZnO calcined at 650 °C.

The influence of ZnO calcination temperature on the microstructural evolution of the CS–GO–ZnO composite fillers was investigated via FE-SEM analysis, as depicted in Figure 3. Pronounced variations in surface topography, particle distribution, and microstructural compactness are observed among the three samples, confirming that the thermal pretreatment of the inorganic phase plays a decisive role in regulating the macromolecular interfacial interactions. Figure 3a,b illustrates the hybrid composite synthesized using uncalcined ZnO. At both low and high magnifications, the microstructure exhibited a highly porous, irregular morphology characterized by interconnected voids and loosely packed domains. This poorly consolidated network is primarily attributed to residual organic species and incomplete decomposition of precursor compounds on the uncalcined ZnO surfaces, which sterically hinder effective interfacial bonding between the ZnO nanoparticles, GO sheets, and the CS matrix [27]. Furthermore, the lack of thermal stabilization allows volatile components to escape during the subsequent film-drying phase, generating micro-voids that disrupt the continuity of the polymer network and form low-resistance pathways for corrosive media.

In stark contrast, the composite prepared using ZnO calcined at the optimized temperature of 500 °C (Figure 3c,d) displayed a remarkably dense, continuous, and homogeneous morphology with a dramatic reduction in visible porosity. The ZnO nanoparticles are uniformly anchored and distributed throughout the CS/GO matrix, with minimal phase separation. Calcination at 500 °C successfully eliminated surface impurities while maintaining an adequate active surface area, thereby maximizing hydrogen bonding and electrostatic coordination between the localized zinc sites and the oxygenated/nitrogenous functional groups of GO and CS. This highly integrated, compact configuration is crucial for barrier applications, as it tortures the diffusion pathways of water, dissolved oxygen, and chloride ions through the cross-linked polymeric matrix. However, increasing the calcination temperature further to 650 °C (Figure 3e,f) induces substantial particle agglomeration and structural heterogeneity. The higher thermal energy at 650 °C promoted accelerated grain growth and partial sintering, leading to the formation of rigid, large-scale ZnO aggregates, as clearly visible near the wrinkled carbonaceous sheets in Figure 3f [28]. This severe agglomeration drastically reduces the net interfacial surface area available for macromolecular anchoring, thereby weakening the filler-matrix adhesion. These localized clusters act as stress concentration zones and create microstructural defects or interfacial micro-gaps around the aggregates, thereby deteriorating the structural integrity of the composite. The morphological observations correlate strongly with the FTIR and XRD findings, validating that a calcination threshold of 500 °C provided an ideal balance between filler crystallinity, uniform spatial dispersion, and robust interfacial compatibility. This optimized microstructure is highly favorable for achieving superior barrier performance and extended corrosion mitigation within the final epoxy composite coating.

### 3.2. Assessment of Coating Performance

#### 3.2.1. Surface Wettability Assessment

**Figure 4:**
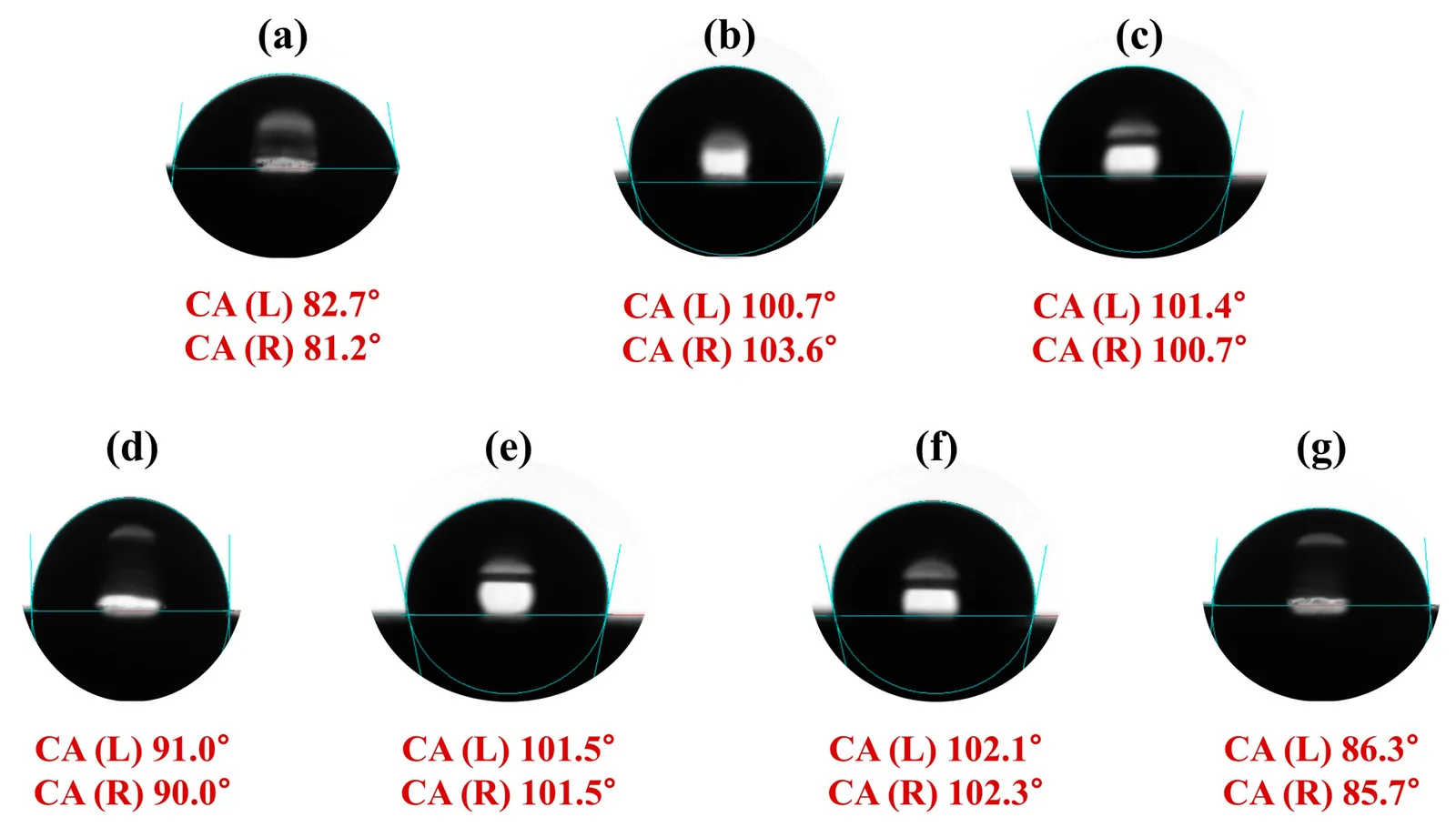
Representative water contact angle (WCA) profiles and droplet geometries on the surfaces of the fabricated epoxy composite coatings. (**a**) GO/EP, (**b**) ZnO/EP (uncalcined), (**c**) ZnO/EP (500 °C), (**d**) ZnO/EP (650 °C), (**e**) CS–GO–ZnO/EP (uncalcined), (**f**) CS–GO–ZnO/EP (500 °C), and (**g**) CS–GO–ZnO/EP (650 °C). The corresponding left (L) and right (R) contact angles are specified for each formulation.

Surface wettability is critical for the long-term effectiveness of protective polymer coatings. This is because a highly hydrophobic surface reduces the initial contact area and thermodynamic wetting between the aggressive electrolyte and the coating matrix. To assess this property, water contact angle (WCA) measurements were performed on the different epoxy composite formulations. The corresponding droplet profiles are depicted in Figure 4, and the average WCA values compiled from the left (L) and right (R) angles are summarized in Table 1. As shown in Figure 4a, the GO/EP coating exhibited the lowest average contact angle (82.0°), indicating a more hydrophilic character than the modified formulations. This behavior is fundamentally ascribed to the abundance of residual oxygen-containing functional groups, such as hydroxyl, epoxy, and carboxyl groups, retained on the GO sheets. These polar groups possess a strong thermodynamic affinity toward water molecules, forming extensive hydrogen-bonding networks at the droplet interface that promote spreading and rapid surface wetting. Interestingly, the uncalcined coatings, ZnO/EP (uncalcined) and CS–GO–ZnO/EP (uncalcined), initially displayed remarkably high contact angles of 102.2° and 101.5°, respectively (Figure 4b,e). While these values superficially suggest high surface hydrophobicity, they represent a transient initial surface state rather than a stable, long-term barrier. In these raw configurations, apparent hydrophobicity is artificially induced by volatile organic residues and incompletely decomposed precursor fragments left behind during low-temperature synthesis. These surface-bound contaminants alter the local surface free energy and temporarily trap micro-air pockets beneath the water droplet. However, upon exposure to an aggressive aqueous environment, these unstable organic fragments undergo rapid hydrolytic dissolution and chemical leaching [29,30]. The elimination of these species collapses the air-trapping pockets, triggers structural degradation, and creates interconnected micro-void paths that allow immediate electrolyte infiltration.

For the neat ZnO-incorporated formulations, thermal calcination at 500 °C yielded a stable, high contact angle of 101.1° (Figure 4c). This performance optimization stems from two interconnected phenomena: the complete thermal desorption of organic impurities yields clean, crystalline nanoparticles that establish a rigid, uniform nanoscale roughness capable of maintaining a true, stable Cassie-Baxter wetting state [31]. At the same time, these cleaned surface sites promote highly effective chemical cross-linking with the surrounding EP matrix, thereby increasing network density and reducing structural flaws that could allow water ingress [32]. Conversely, elevating the filler calcination threshold to 650 °C severely degraded the hydrophobic quality of the binary composite, reducing the average contact angle to 90.5° (Figure 4d). This drop-off is driven by intense thermal sintering and accelerated grain-boundary growth in the ZnO nanoparticles at temperatures exceeding 600 °C [9,33]. The formation of massive, rigid aggregates drastically flattens the required nanoscale texturing, eliminating the air-trapping microstructures while simultaneously generating severe internal stress concentrations and localized micro-fractures. Crucially, the ternary hybrid coating prepared at the optimized calcination temperature, CS–GO–ZnO/EP (500 °C), achieved the highest average contact angle of 102.2° with a highly symmetric droplet profile (Figure 4f). The exceptional hydrophobic stability of this specific configuration is driven by a strong structural synergism among three components: the inherently low surface energy of the cross-linked bulk epoxy matrix, the controlled nanoscale roughness provided by the well-dispersed 500 °C-calcined ZnO nanoparticles, and the 2D wrinkled geometry of the GO sheets, which creates a highly tortuous, hierarchical surface architecture.

CS effectively acts as a macromolecular bridge, seamlessly linking the inorganic ZnO and carbonaceous GO phases into a densely integrated hybrid network with minimized surface free energy. This optimized surface topography works in perfect harmony with the high coating resistance (*R_f_*) observed in the EIS analysis, proving that robust surface hydrophobicity directly delays electrolyte wetting and acts as the primary defense mechanism against aggressive ion transport [34,35,36]. Conversely, the CS–GO–ZnO/EP (650 °C) composite coating experienced a substantial decline in hydrophobic character, yielding a contact angle of only 86.0° (Figure 4g). As evidenced by the increase in electrochemical capacitance parameters, severe thermal agglomeration and phase sintering of the ZnO nanoparticles at 650 °C completely disrupt the uniform ternary dispersion. The resulting microstructural collapse produces a highly heterogeneous, porous, and defect-rich surface layer. Capillary forces within these expanded surface pores drive a rapid transition toward a hydrophilic state, rendering the underlying carbon steel substrate highly susceptible to localized corrosion. The distinction between initial surface wettability and long-term barrier performance provides critical insights into the degradation mechanisms of these hybrid polymeric coatings.

As summarized in Table 1, the uncalcined ternary composite, CS–GO–ZnO/EP (uncalcined), initially demonstrated a high mean contact angle of 101.5°. This high value, combined with a negligible standard deviation, indicates an initially symmetric and highly reproducible hydrophobic surface topography. However, as corroborated by the long-term EIS analysis, this uncalcined sample exhibited a very low *R_ct_* over extended exposure periods. This stark contradiction underlines a fundamental divergence between a material’s transient surface hydrophobicity and its ability to maintain barrier properties over time under operational conditions. In the case of the uncalcined composite, the initial hydrophobic behavior is artificially driven by volatile organic residues and amorphous phases remaining on the raw, untreated ZnO surfaces. These impurities induce a temporary surface roughening that physically supports the water droplet in a metastable state. Once exposed to a continuous electrolytic environment, these surface defects and unstable organic fragments undergo rapid hydrolytic dissolution. This degradation accelerates localized delamination and interlayer separation between the CS–GO framework and the underlying epoxy matrix [30], destroying the protective barrier network and facilitating fast electrolyte pathing.

In contrast to the stable hydrophobic performance observed at 500 °C, the CS–GO–ZnO/EP formulation prepared at 650 °C displayed the lowest average contact angle (86.0°) among all evaluated ternary hybrid coatings. This represents a substantial drop-off in hydrophobic character compared to the optimized 500 °C sample. This severe loss of hydrophobicity is primarily a consequence of the thermal sintering and degradation of the particulate inorganic ZnO phase at elevated temperatures. At 650 °C, excessive thermal energy drives intense particle aggregation, as supported by the sharp increase in the system’s electrochemical capacitance parameters (which typically signal increased water absorption and a larger electrochemically active surface area). Rather than maintaining a uniform nano-roughness, the sintered ZnO nanoparticles form massive, localized clusters. This severe particle aggregation creates a highly irregular, porous, and defect-rich surface profile. Due to the high density of micro-cracks and open capillaries around these aggregates, capillary action forces rapid water absorption into the coating. This shifts the surface behavior from hydrophobic to a highly hydrophilic state, destroying the coating’s ability to resist electrolyte wetting.

#### 3.2.2. Adhesion Strength Evaluation

**Figure 5:**
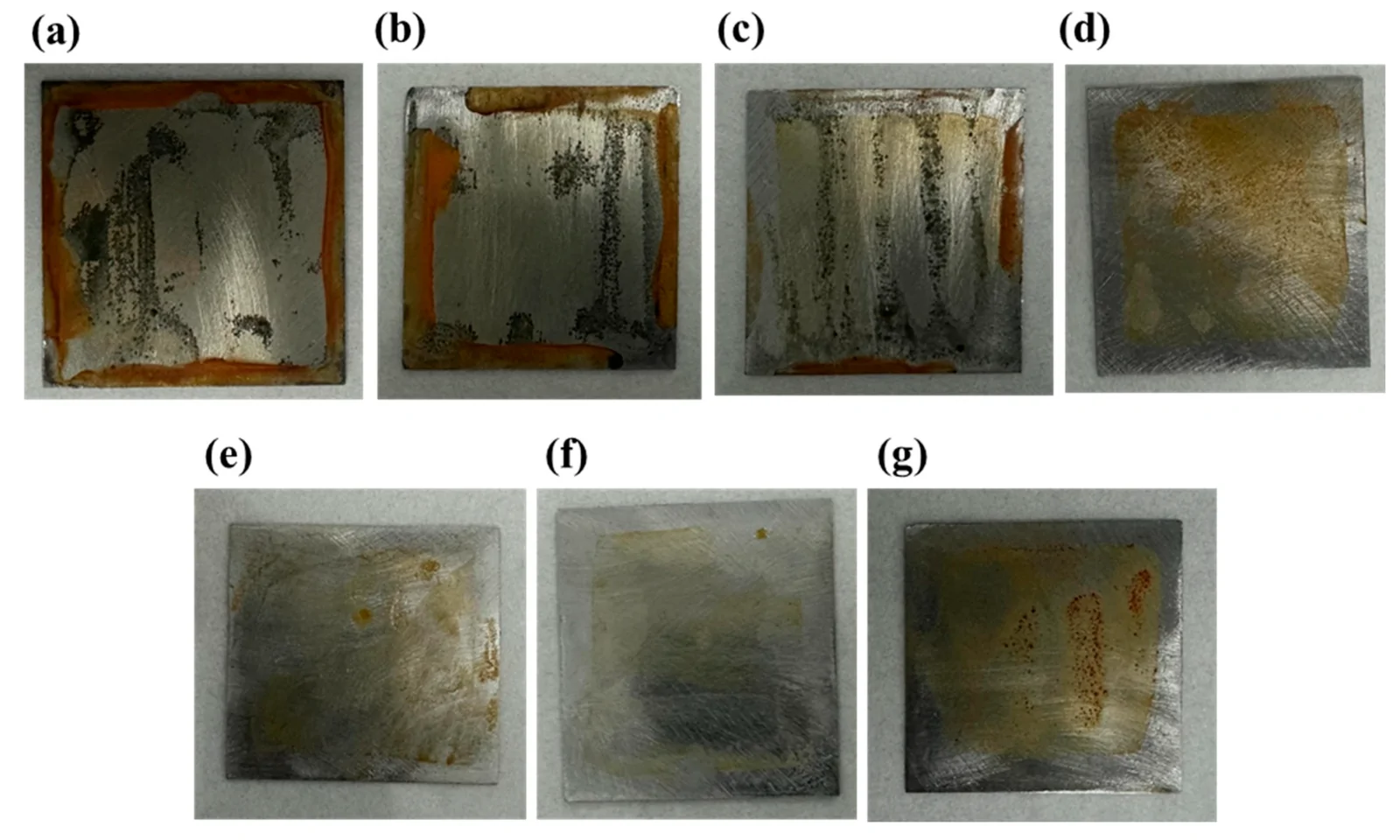
Optical photographs of the carbon steel surfaces after pull-off testing following 120 h of immersion in a 3.5 wt.% NaCl aqueous solution, showcasing the visual extent of rust formation and sub-film corrosion across the different coating systems. (**a**) GO/EP, (**b**) ZnO/EP (uncalcined), (**c**) ZnO/EP (500 °C), (**d**) ZnO/EP (650 °C), (**e**) CS–GO–ZnO/EP (uncalcined), (**f**) CS–GO–ZnO/EP (500 °C), (**g**) CS–GO–ZnO/EP (650 °C).

The adhesion capacity of a protective polymeric coating on a metallic surface is a primary parameter governing its long-term durability and resistance against localized corrosion. Inadequate interfacial adhesion facilitates the capillary ingress of aggressive electrolytes along the coating/substrate boundary, accelerating underfilm corrosion, osmotic blistering, and premature catastrophic delamination [31]. To evaluate this property, both the initial “dry” pull-off adhesion strength and the post-immersion “wet” pull-off adhesion strength (after a 120 h exposure period to a 3.5 wt.% NaCl aqueous solution) were measured in accordance with ASTM D4541 standards. The quantitative adhesion metrics are summarized in Table 2. Furthermore, representative optical photographs of the detached carbon steel coupon interfaces following the 120 h immersion test are presented in Figure 5, serving as visual indicators of rust formation, active pitting, and the overall degree of interfacial degradation.

Among all investigated formulations, the ternary hybrid coating prepared at the optimized calcination temperature, CS–GO–ZnO/EP (500 °C), demonstrated the highest dry adhesion strength (7.794 MPa) and wet adhesion strength (7.648 MPa). These values exceed the standard industrial benchmark of 5 MPa, indicating exceptionally robust and hydrolytically stable bonding forces between the hybrid composite coating and the underlying carbon steel substrate. Three synergistic structural phenomena drive this prominent adhesion enhancement. First, the optimal calcination temperature of 500 °C produces highly crystalline ZnO nanoparticles with pristine surfaces free of blocking organic contaminants, thereby maximizing the direct formation of strong Fe–O–Zn covalent linkages at the coating/steel interface [37]. Second, the abundant amine (–NH_2_) and –OH functional groups distributed along the backbones of the co-intercalated CS molecules actively participate in strong hydrogen bonding and coordination-covalent linkages with the localized iron atoms on the steel surface, promoting pronounced chemisorption [38]. Lastly, the 2D wrinkled topology of GO provides oxygenated networks (–COOH, –OH, and C–O–C) that maximize mechanical interlocking while acting as secondary macromolecular anchoring spots. This constructs a highly dense, cross-linked hybrid network with a maximized interfacial contact area [39]. Concurrently, the binary ZnO/EP (500 °C) formulation demonstrated the highest relative adhesion retention of 99.1% across all test blocks. While its baseline dry adhesion strength was modest (4.655 MPa), the coating exhibited remarkable stability against electrolyte-driven delamination. The well-crystallized ZnO fillers establish an impermeable physical barrier network that shields the interface from corrosive species over extended timescales [40], which suppresses hydrolytic degradation at the polymer/steel junction. Conversely, despite exhibiting a high initial dry adhesion value (6.632 MPa), the uncalcined binary ZnO/EP sample retained only 78.6% of its mechanical integrity after immersion. This significant loss indicates that residual organic fragments and unstable precursor phases on the uncalcined ZnO surfaces decompose slowly upon hydration, creating pathways that weaken interfacial cohesion.

As noted in Table 2, the optimized ternary CS–GO–ZnO/EP (500 °C) composite coating retained an exceptional wet adhesion strength of 7.648 MPa, marking the highest absolute post-immersion value among all formulations. While a minor drop of 0.146 MPa was detected between the dry and wet states due to trace interfacial relaxation or localized moisture uptake, the absolute value remained vastly superior to the rest of the group. This high stability is directly linked to the highly tortuous, hydrophobic cross-linked structure of the CS–GO–ZnO/EP (500 °C) filler configuration, which successfully retards electrolyte diffusion toward the underlying steel, thereby preserving the primary covalent and hydrogen-bonded networks [41]. In stark contrast, although the over-calcined CS–GO–ZnO/EP (650 °C) composite maintained a high mathematical retention percentage (94.4%), it yielded critically deficient dry (3.654 MPa) and wet (3.451 MPa) adhesion values. This severely compromised adhesive performance is directly traceable to the excessive thermal sintering and large-scale particle aggregation of ZnO at 650 °C. The resulting macro-aggregates disrupt the uniformity of the polymer matrix, lowering the density of active anchoring sites at the metal boundary and creating internal stress concentrations that easily prompt interfacial detachment. Finally, the neat GO/EP coating demonstrated the lowest wet adhesion strength (4.243 MPa) and the poorest adhesion retention (70.2%). This rapid degradation is driven by the highly hydrophilic nature of unhybridized GO sheets; intense water absorption triggers swelling of the GO structures, inducing strong localized internal shear stresses at the polymer/substrate boundary that accelerate spontaneous underfilm delamination [42].

#### 3.2.3. Surface Topography

**Figure 6:**
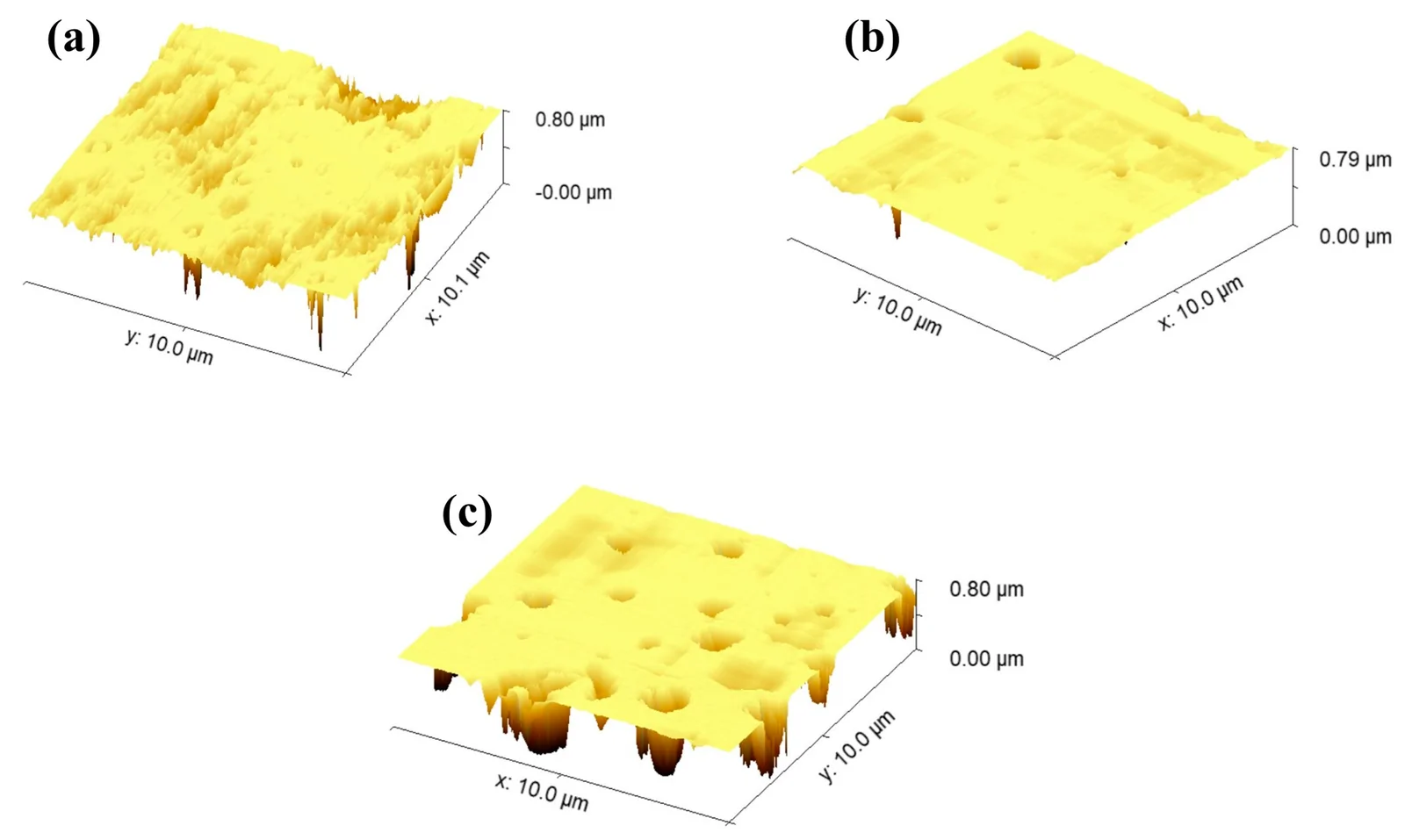
3D AFM surface topography profiles (10 μm × 10 μm) of the fabricated ternary hybrid coatings: (**a**) CS–GO–ZnO/EP (uncalcined), (**b**) CS–GO–ZnO/EP (500 °C), and (**c**) CS–GO–ZnO/EP (650 °C).

To gain a deeper understanding of how the internal microstructure influences surface properties, AFM was employed to map the 3D surface topographies and nanoscale roughness configurations of the composite coatings. The resulting 3D AFM topographical profiles for the ternary hybrid formulations prepared with uncalcined, 500 °C-calcined, and 650 °C-calcined ZnO are illustrated in Figure 6a–c, respectively. As revealed in Figure 6a, the CS–GO–ZnO/EP coating synthesized with uncalcined ZnO possesses a highly defective, disordered surface morphology characterized by numerous micro-voids, shallow depressions, and loose structural plateaus. The absence of sharp or well-defined crystalline asperities in this 3D profile indicates the presence of volatile organic remnants and unreacted precursor phases carried over from the raw ZnO synthesis. These carbonaceous impurities sterically restrict the spatial dispersion and cross-linking efficiency of the ZnO nanoparticles within the macromolecular CS–GO framework [43]. Quantitatively, this coating exhibited a mean roughness (Sa) of 43.88 ± 4.2 nm and an RMS roughness (Sq) of 69.07 ± 5.2 nm, with a high maximum height (Sz = 911.7 nm) and deep surface pits (Sv = 763.6 nm) as tabulated in Table 3. Interestingly, despite this deficient surface quality, the coating initially recorded a high-water contact angle of 101.5°. The AFM topography explained this phenomenon: the micro-depressions and localized surface cavities physically trap air pockets beneath the placed droplet, initiating a metastable Cassie–Baxter state. However, because these sub-surface regions are composed of unstable, uncalcined organic fractions, continuous exposure to moisture prompts rapid hydrolytic dissolution. This causes the trapped air layer to collapse, driving a transition to a fully wet state that accelerates the interfacial failure detected during the electrochemical evaluations.

In sharp contrast, the ternary composite coating incorporating ZnO calcined at 500 °C (Figure 6b) displayed a drastically altered topographical fingerprint. The mapped surface is exceptionally compact, continuous, and dense, with a near-complete elimination of observable surface pores, macro-voids, or localized depressions. The 3D profile revealed a highly uniform distribution of subtle nanoscale asperities across the entire scanned area (10 μm × 10 μm), confirming that the ZnO nanoparticles remained highly decoupled from one another and evenly dispersed within the cross-linked polymeric matrix. This optimal surface texture is quantitatively supported by the lowest mean roughness (Sa = 34.30 ± 3.8 nm) among all formulations, along with the lowest surface slope (Sdq = 1.040 ± 0.09) and the smallest maximum peak height (Sp = 25.9 nm). The reduced roughness and uniform surface profile work in tandem with the low surface energy of the epoxy-CS backbone to form a robust, stable air-trapping boundary. The AFM data confirmed that thermal pretreatment at 500 °C successfully purifies the ZnO surface sites, allowing them to establish strong interfacial chemical coordination with the surrounding polymer chains. This dense matrix integration explains why the 500 °C coating consistently achieved superior pull-off adhesion strength and exceptional long-term electrochemical barrier protection.

Conversely, the AFM analysis of the over-calcined CS–GO–ZnO/EP (650 °C) coating revealed severe structural degradation and high surface roughness heterogeneity (Figure 6c). The surface is dominated by large, irregularly shaped rigid protrusions that are separated by wide, deep macro-valleys and open interfacial cracks. This highly disrupted topography is quantitatively confirmed by the highest roughness values recorded (Sa = 71.1 ± 5.6 nm and Sq = 131.7 ± 8.5 nm) and the highest surface slope (Sdq = 1.476 ± 0.14) as tabulated in Table 3. This occurs because excessive thermal exposure at 650 °C drives intense nanoparticle sintering and rapid grain boundary growth, forcing the inorganic phase to collapse into large macro-aggregates. These massive crystalline clusters cannot maintain uniform dispersion within the polymer phase; instead, they undergo phase separation and migrate toward the surface, generating massive internal stress concentration zones that induce micro-cracking across the matrix. Consequently, the surface water contact angle drops sharply to 86.0°, falling below the fundamental hydrophobic threshold (<90°). The open, interconnected cracks and exposed hydrophilic ZnO aggregates mapped by the AFM tips act as high-energy capillary channels, wicking the electrolytic solution deep into the coating layer and directly leading to the rapid corrosion failure of the underlying steel substrate.

### 3.3. Electrochemical Evaluation

#### 3.3.1. Potentiodynamic Polarization (Tafel) Analysis

**Figure 7:**
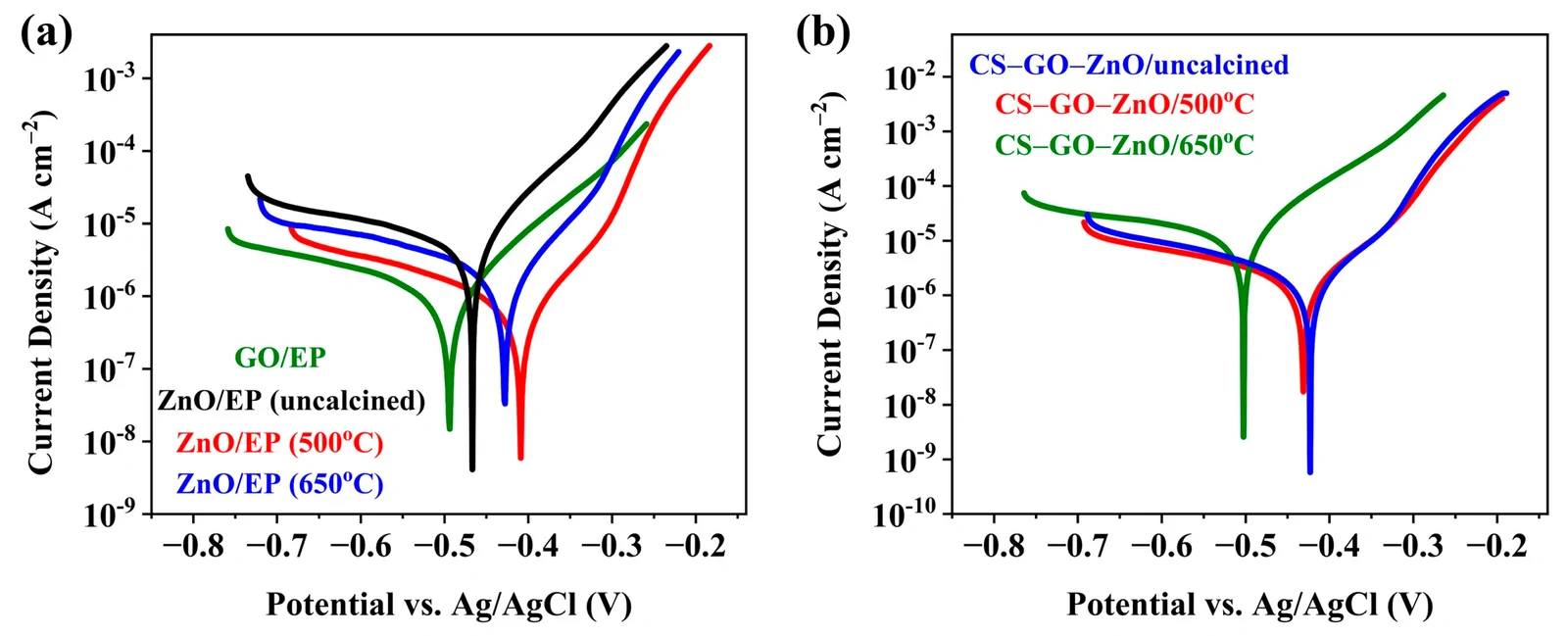
Potentiodynamic polarization (Tafel) curves for the coated carbon steel substrates recorded after 120 h of immersion in a 3.5 wt.% NaCl solution. (**a**) Binary GO/EP and ZnO/EP coatings prepared at different ZnO calcination temperatures. (**b**) Ternary hybrid CS–GO–ZnO/EP composite coatings as a function of filler calcination conditions.

Potentiodynamic polarization measurements were performed to evaluate the instantaneous kinetics of the corrosion processes occurring at the coating/substrate interface after a prolonged 120 h immersion period in 3.5 wt.% NaCl. The polarization curves for the binary formulations, ZnO/EP (uncalcined, 500 °C, and 650 °C) and GO/EP, are displayed in Figure 7a. In contrast, the curves for the ternary hybrid composite coatings (CS–GO–ZnO/EP) are presented in Figure 7b. The calculated electrochemical kinetic parameters, including corrosion potential (E_corr_), corrosion current density (I_corr_), anodic/cathodic Tafel slopes (β_a_/β_c_), corrosion rate (CR), and polarization resistance (R_p_), are compiled in Table 4. As detailed in Table 4, the binary ZnO/EP coating formulated with 500 °C-calcined nanoparticles exhibited the lowest I_corr_ (1.73 μA cm^−2^) and a minimal CR (0.020 mm/y), representing a 78.5% reduction in corrosion kinetics compared to the raw ZnO/EP (uncalcined) counterpart (8.07 μA cm^−2^). This enhanced performance is attributed to the high crystallinity and organic-free surfaces achieved at the optimal calcination temperature, which facilitates a uniform particle dispersion and cross-linking density within the bulk epoxy matrix [44]. This configuration yields a dense physical barrier that impedes the pathing of aggressive ions. The neat GO/EP coating demonstrated moderate protection efficiency (I_corr_ = 2.29 μA cm^−2^), stemming from the high aspect ratio of the GO layers that forces a tortuous diffusion pathway for penetrating molecules [45]. Conversely, increasing the calcination temperature to 650 °C caused the Icorr to surge to 4.20 μA cm^−2^, indicating that structural sintering and particle aggregation led to internal void propagation and degraded barrier capabilities.

For the ternary systems (Table 4 and Figure 7b), the thermal history of the embedded filler exerted powerful control over the resulting coating performance. The ternary hybrid CS–GO–ZnO/EP (500 °C) composite achieved the highest polarization resistance (R_p_ = 7.71 kΩ) among all evaluated ternary matrices. Crucially, this optimized sample yielded an exceptionally high anodic Tafel slope (β_a_ = 522.280 mV dec^−1^), indicating a strong suppression of the anodic dissolution reaction of Fe from the underlying carbon steel substrate. This significant anodic inhibition is visually obvious in Figure 7b, where the curve at 500 °C maintained a highly suppressed current density profile across the active anodic domain. This behavior verifies that the pristine, well-dispersed ternary components form a highly stable, integrated passive network that restricts the activation of corrosion sites.

A careful examination of the Tafel data revealed that the absolute position of the polarization curve on the current density axis does not always directly correspond to the extrapolated I_corr_ value when the anodic and cathodic Tafel slopes differ significantly. The intersection point of the anodic and cathodic Tafel extrapolation lines determines the I_corr_. For CS–GO–ZnO/EP (500 °C), the exceptionally high anodic Tafel slope (β_a_ = 522.28 mV dec^−1^) strongly suppresses anodic dissolution, while the intersection of the anodic and cathodic extrapolated lines yields the reported I_corr_ of 6.428 μA cm^−2^. The uncalcined sample, with lower Tafel slopes (β_a_ = 267.26 mV dec^−1^, β_c_ = 106.35 mV dec^−1^), yields an intersection point at 4.64 μA cm^−2^ despite the curve appearing higher on the plot. This phenomenon is well-documented in corrosion science, where Tafel slope variations can lead to apparent discrepancies between curve position and extrapolated I_corr_ [46,47]. In contrast, the over-calcined CS–GO–ZnO/EP (650 °C) matrix suffered catastrophic barrier degradation, marked by a massive increase in Icorr to 27.56 μA cm^−2^, a severe drop in polarization resistance down to 1.21 kΩ, and a high corrosion rate of 0.320 mm/y. The cathodic curve for the 650 °C composite is shifted significantly toward higher current densities, indicating an accelerated oxygen reduction rate at the metal boundary. This accelerated degradation highlights the detrimental effects of uncontrolled thermal sintering. At 650 °C, the destruction of the uniform ternary dispersion and the creation of large-scale ZnO aggregates generate extensive mechanical micro-cracking and high phase-boundary heterogeneity within the polymer sheet [48]. These structural defects act as open capillary networks that rapidly channel the corrosive media to the underlying steel, causing severe barrier failure.

#### 3.3.2. Electrochemical Impedance Spectroscopy (EIS)

**Figure 8:**
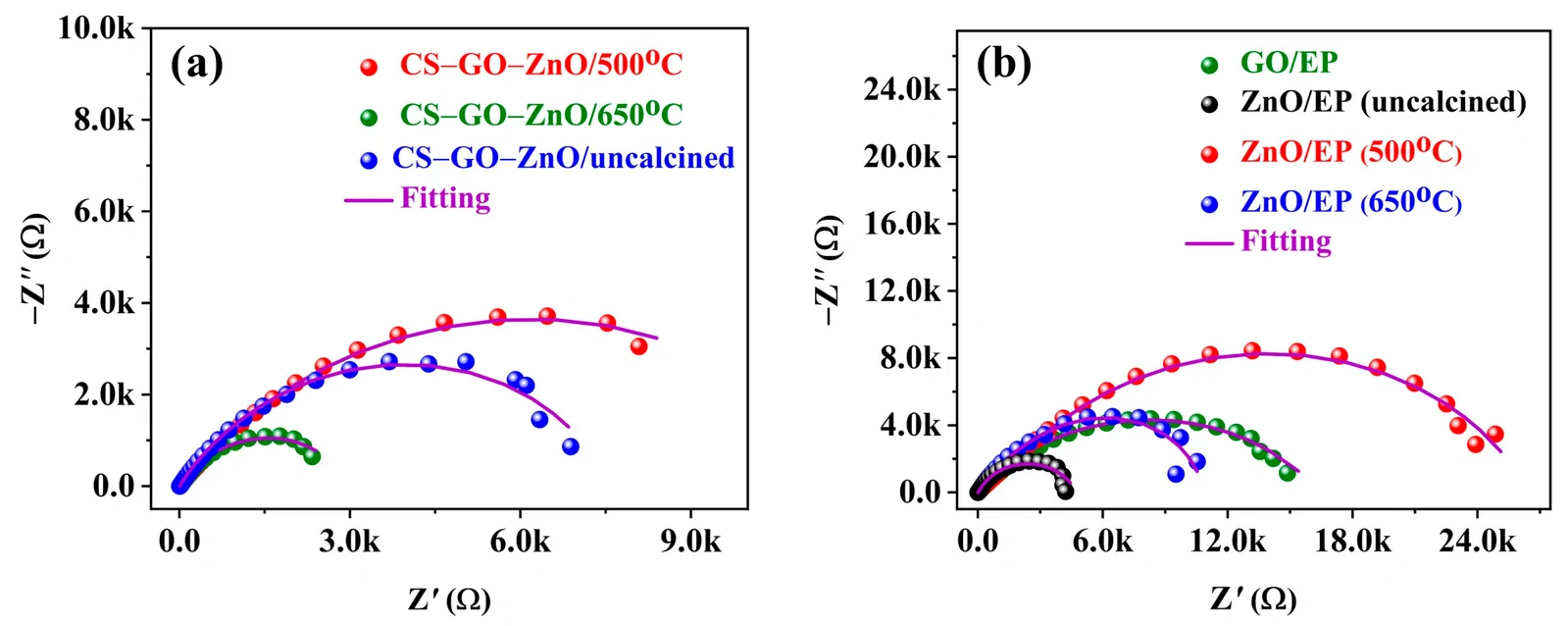
Nyquist impedance profiles recorded for the coated carbon steel substrates after 120 h of immersion in a 3.5 wt.% NaCl aqueous solution. (**a**) Binary GO/EP and ZnO/EP formulations prepared at different filler calcination temperatures. (**b**) Ternary hybrid CS–GO–ZnO/EP composite coatings at different filler calcination temperatures.

EIS was employed to thoroughly investigate the long-term barrier mechanisms, coating degradation profiles, and underlying interfacial kinetics of the modified epoxy matrices after a 120-h immersion period in a 3.5 wt.% NaCl solution. The resulting Nyquist plots for the binary (GO/EP and ZnO/EP) and ternary hybrid (CS–GO–ZnO/EP series) composite coatings are illustrated in Figure 8. The impedance responses typically manifest as depressed capacitive semicircles spanning the high-to-low frequency domains, where the geometric diameter of the loop correlates directly with the coating resistance (R_coat_) and the charge transfer resistance (*R_ct_*). To quantitatively evaluate the individual electrochemical contributions, the raw impedance data were modeled using equivalent electrical circuits.

The optimized fit parameters are compiled in Table 5. As shown in the Table, the binary ZnO/EP coating prepared with 500 °C-calcined nanoparticles displayed the largest *R_ct_* (2.43 × 10^5^ Ω), indicating superior protection against active electrochemical reactions at the carbon steel interface. The corresponding double-layer capacitance descriptor (CPE_c_) was significantly lower (29.9 μΩ^−1^ cm^−2^ s) than that of the other binary samples, with an exponent approaching unity (n_c_ = 0.74), confirming a uniform coating layer with low localized capacitance. For the uncalcined ZnO/EP, a higher *R_coat_* (3.47 × 10^4^ Ω) stood in contrast to its low *R_ct_* (4.36 × 10^3^ Ω). This demonstrates that while the uncalcined particles provide a moderate initial physical barrier, they fail to block charge transfer at the metal boundary once moisture reaches the interface. In comparison, the neat GO/EP exhibited the lowest *R_ct_* (298 Ω), representing the lowest protection efficiency against corrosive attack due to its unshielded hydrophilic channels. The binary ZnO/EP prepared with 650 °C-calcined fillers showed a significantly lower *R_ct_* (4.95 × 10^3^ Ω) along with anomalous n values, confirming that high-temperature particle sintering introduces microstructural pathways that compromise the bulk epoxy matrix.

A notable anomaly appears within the ternary hybrid series. The uncalcined CS–GO–ZnO/EP coating recorded an exceptionally low *R_ct_* (2.97 Ω), despite displaying a relatively low corrosion current density (I_corr_ = 4.64 μA cm^−2^) during potentiodynamic polarization testing. This apparent divergence stems from the different nature of the two methods. Tafel sweeps measure transient current responses under rapidly changing applied overpotentials, whereas EIS evaluates the steady-state equilibrium properties of the interface across a wide frequency range. This makes EIS a more reliable indicator of long-term barrier performance and network stability [49]. The low *R_ct_* value indicates that the uncalcined composite fails to establish a continuous barrier layer, leading to ion conduction through open micropores and curing defects. This disruption in the epoxy cross-linking density is caused by volatile organic residues remaining on the uncalcined ZnO nanoparticles, which generate preferred pathways for electrolyte ingress [50]. Conversely, the ternary CS–GO–ZnO/EP coating calcined at the optimized temperature of 500 °C demonstrated a significantly enhanced barrier response, achieving a high coating film resistance (*R_coat_* = 1.06 × 10^5^ Ω) and a stable charge transfer resistance (*R_ct_* = 9.37 × 10^3^ Ω). The balanced CPE_of_ and CPE_c_ values, along with their stable exponents (0.80 and 0.62), indicate a uniform, low-porosity configuration. At this optimal thermal threshold, the ZnO nanoparticles achieved a balance between high crystallinity and controlled particle size, facilitating an even spatial dispersion within the CS–GO framework. This high crystallinity supports a structurally uniform inorganic-organic hybrid network. Beyond acting as a physical barrier, these optimized ZnO nanoparticles function as efficient sacrificial inhibitors. The controlled dissolution of these structures releases Zn^2+^ ions into the penetrating electrolyte. These ions migrate toward cathodic sites on the steel substrate, reacting to deposit a tightly adherent passive layer of zinc hydroxide or zinc chloride [51]. This passive layer physically isolates the substrate, effectively inhibiting both the anodic dissolution of iron and the cathodic oxygen reduction reaction.

When the calcination temperature is raised to 650 °C, the ternary CS–GO–ZnO/EP composite undergoes a severe decline in performance, with the coating resistance dropping sharply to 1.65 × 10^3^ Ω and the charge transfer resistance falling to 1.24 × 10^3^ Ω. This structural failure highlights the adverse effects of high-temperature thermal treatment. Calcination at 650 °C drives intense particle sintering and grain boundary growth, causing the ZnO nanoparticles to collapse into large macro-aggregates. This severe agglomeration reduces the effective interfacial surface area available for binding with the surrounding CS–GO framework, leading to increased pore volume and greater network porosity, as indicated by the elevated CPE values and the anomalous exponent (n = 1.1) [52]. Furthermore, this excessive thermal treatment produces large, highly crystalline particles that exhibit reduced solubility in the micro-electrolyte environment. This lowers the release rate of active Zn^2+^ inhibitors, weakening the system’s ability to deposit a protective passive film on the steel substrate [53]. Similarly, the uncalcined ZnO particles, distorted by residual organic species, poor crystallinity, and an irregular morphology, exhibit unpredictable dissolution kinetics and poor binding compatibility with the CS–GO framework [54]. This highlights that a thermal pretreatment threshold of 500 °C is crucial for achieving optimal barrier properties and sustained corrosion protection.

### 3.4. Proposed Corrosion Protection Mechanism

An intricate, multi-tiered protection mechanism governs the superior anticorrosion performance of the optimized ternary hybrid composite coating. This performance relies on the strong structural and chemical synergism established among the three distinct fillers, CS, GO, and optimally calcined ZnO nanoparticles, dispersed within the cross-linked EP matrix. The overall protection framework operated via three primary, simultaneous pathways: interfacial chemisorption, tortuous physical barrier execution, and active cathodic passivating inhibition.

CS served as the foundational structural modifier and interface bridge within the composite. The extensive distribution of active –NH_2_ and –OH functional groups along its polymeric backbone facilitated strong coordination-covalent bonding and heavy chelation with the Fe atoms on the carbon-steel substrate surface. This promoted an exceptionally high density of stable chemisorption sites, significantly boosting pull-off adhesion strength and creating a highly durable, hydrolytically stable interfacial boundary that resists underfilm electrolyte migration.

GO introduced high-aspect-ratio, 2D carbonaceous planes into the formulation. When thoroughly exfoliated and integrated, these aligned GO sheets act as a highly efficient physical barrier network within the EP cross-links. This structure establishes a highly tortuous geometric diffusion pathway (the “maze effect”), forcing penetrating water molecules, dissolved oxygen, and aggressive chloride ions (Cl^−^) to travel along a significantly extended path length [55,56]. This delay retards the arrival of corrosive species at the reactive metal interface.

ZnO acted as the active thermodynamic and chemical inhibitor within the coating. Upon micropore infiltration by an invading aqueous electrolyte, the embedded ZnO phase undergoes controlled, localized dissolution to release active Zn^2+^ ions. These migrating Zn^2+^ species move toward high-pH cathodic zones on the steel substrate, where they react with OH^−^ ions generated during oxygen reduction to form insoluble Zn(OH)_2_ and other zinc-containing corrosion products. Previous studies employing XPS analysis have confirmed the formation of Zn(OH)_2_, ZnO, and zinc-based passive layers resulting from Zn^2+^ migration and precipitation processes, which effectively block active corrosion sites and enhance the barrier properties of protective coatings [3,57]. Consequently, the resulting passive film acts as a secondary protective barrier that seals galvanic sites and suppresses further corrosion propagation.

The inorganic filler’s thermal history during preprocessing dictates whether these three protection modes can operate effectively. The physical state of the filler changed significantly across three distinct thermal conditions. The uncalcined ZnO particles are bounded by volatile organic impurities, processing chemical remnants, and disordered, amorphous outer surfaces. These shielding organic compounds physically obstruct proper interfacial cross-linking and interrupt the curing network of the surrounding EP matrix, giving rise to localized micro-voids, high internal porosity, and irregular surface depressions. While the resulting surface roughness can initially trap air pockets to yield a false, transient hydrophobic water contact angle (101.5°), the structural defects rapidly degrade under water exposure. This induces premature hydrolytic delamination, layer separation, and complete loss of barrier protection.

Calcination at 500 °C represented the optimal thermal window where physical barrier performance, chemical adhesion, and active inhibition are maximized simultaneously [56]. Thermal treatment at this temperature eliminates shielding organic contaminants, yielding highly crystalline, pure ZnO nanoparticles with uniform spatial dimensions. These clean surfaces facilitate strong hydrogen bonding and chemical cross-linking with the co-intercalated CS–GO, establishing a highly homogeneous, dense hybrid network. Topographically, this optimized dispersion produced a stable nanoscale roughness that maintained a robust Cassie-Baxter state, resulting in a high water contact angle of 102.2° and excellent resistance to electrolyte wetting. Electrochemically, this configuration maintained an ideal balance of uniform particle distribution and controlled solubility, enabling a steady, long-term release of passivating Zn^2+^ inhibitors. These properties work in harmony with delivered high R_p_, an exceptionally high anodic Tafel slope (β_a_ = 522.280 mV dec^−1^) that suppresses Fe dissolution, and excellent pull-off adhesion retention (98.1%) after continuous immersion.

Elevating the calcination threshold to 650 °C severely damages the protective properties of the hybrid composite. At this high temperature, excessive thermal sintering triggers intense particle aggregation and rapid grain growth, collapsing the nanoscale morphology into large macro-clusters. These oversized aggregates cannot maintain a uniform dispersion within the organic matrix, resulting in phase separation, severe coating non-uniformity, and the generation of large internal stress concentrations. This phase disruption prompts the formation of interconnected micro-cracks and open capillary pathways throughout the matrix, lowering the water contact angle to 86.0° and shifting the coating into a hydrophilic state. Furthermore, the massive grain dimensions and reduced effective surface area of the sintered ZnO clusters slow down the dissolution and release kinetics of active Zn^2+^ ions, compromising the system’s ability to deposit a passivating film. This structural breakdown allows aggressive ions to rapidly infiltrate the coating, leading to the corrosion failure of the underlying carbon steel substrate.

## 4. Conclusions

This work established that the thermal history of ZnO nanoparticles is not a secondary processing detail but a decisive factor governing the protective performance of chitosan-graphene oxide/epoxy hybrid coatings. By identifying 500 °C as the optimal calcination window, the study demonstrated that judicious thermal pretreatment transforms ZnO from a passive filler into an active participant in a tripartite protection strategy. At this temperature, the inorganic phase achieved a delicate balance: its surface became chemically accessible for robust interfacial bonding with the polymer matrix, its dispersion remained uniform without succumbing to aggregation, and its electrochemical activity was preserved, enabling sustained release of corrosion-inhibiting Zn^2+^ species. Previous studies have reported, through XPS characterization, that dissolved Zn^2+^ species can precipitate as Zn(OH)_2_/ZnO-containing passive films on cathodic regions, thereby contributing to corrosion suppression and enhanced barrier protection. Although the corrosion protection mechanism proposed in the present work is supported by electrochemical analysis and literature reports, direct characterization of the Zn-containing passive film by XPS, EDS mapping, or corrosion product analysis was beyond the scope of this study. Future work will focus on verifying the composition and evolution of these passive films and clarifying their contribution to long-term corrosion protection. The resulting coating architecture combined the effectiveness of a physical barrier, strong substrate adhesion, and active cathodic passivation into a single, coherent system. Conversely, deviations from this thermal optimum, whether by omitting calcination or by overheating, introduce structural vulnerabilities that undermine long-term coating integrity. These findings challenge the common practice of using ZnO without regard for its thermal preconditioning and provide a valuable design principle for developing environmentally sustainable, high-performance protective coatings for carbon steel in chloride-rich environments.

## Figures and Tables

**Table 1:** Water contact angle values of the fabricated epoxy (EP) composite coatings.

Coating Sample	Average Water Contact Angle (WCA °)
GO/EP	82.0 ± 2.1
ZnO/EP (uncalcined)	102.2 ± 1.8
ZnO/EP (500 °C)	101.1 ± 1.5
ZnO/EP (650 °C)	90.5 ± 2.3
CS–GO–ZnO/EP (uncalcined)	101.5 ± 1.6
CS–GO–ZnO/EP (500 °C)	102.2 ± 1.2
CS–GO–ZnO/EP (650 °C)	86.0 ± 2.5

Values are presented as mean ± standard deviation (n = 3 measurements per sample).

**Table 2:** Dry and wet pull-off adhesion strengths and corresponding retention percentages for the various epoxy-based composite coatings.

Coating Sample	Dry Adhesion Strength (MPa)	Wet Adhesion Strength After 120 h (MPa)	Adhesion Retention (%)
GO/EP	6.046 ± 0.42	4.243 ± 0.38	70.2
ZnO/EP (uncalcined)	6.632 ± 0.45	5.210 ± 0.41	78.6
ZnO/EP (500 °C)	4.655 ± 0.31	4.612 ± 0.29	99.1
ZnO/EP (650 °C)	4.618 ± 0.35	3.643 ± 0.33	78.9
CS–GO–ZnO/EP (uncalcined)	5.070 ± 0.38	4.758 ± 0.36	93.8
CS–GO–ZnO/EP (500 °C)	7.794 ± 0.52	7.648 ± 0.48	98.1
CS–GO–ZnO/EP (650 °C)	3.654 ± 0.28	3.451 ± 0.30	94.4

**Table 3:** Surface roughness parameters extracted from AFM analysis of ternary hybrid coatings.

Parameter	CS–GO–ZnO/EP (Uncalcined)	CS–GO–ZnO/EP (500 °C)	CS–GO–ZnO/EP (650 °C)
RMS roughness, Sq (nm)	69.07 ± 5.2	97.06 ± 6.8	131.7 ± 8.5
Mean roughness, Sa (nm)	43.88 ± 4.2	34.30 ± 3.8	71.1 ± 5.6
Surface slope, (Sdq)	1.449 ± 0.12	1.040 ± 0.09	1.476 ± 0.14

Values are presented as mean ± standard deviation (n = 3 scan areas per sample).

**Table 4:** Electrochemical kinetic parameters extracted from Tafel polarization curves for GO/EP, binary ZnO/EP, ternary hybrid CS–GO–ZnO/EP composite coatings.

Coating Sample	E_corr_ (V)	I_corr_ (μA cm^−2^)	β_a_ (mV dec^−1^)	β_c_ (mV dec^−1^)	CR (mm/y)	Rp (kΩ)
GO/EP	−0.494	2.29	285.33	105.52	0.027	14.59
ZnO/EP (uncalcined)	−0.466	8.07	165.00	66.90	0.093	2.56
ZnO/EP (500 °C)	−0.410	1.73	227.84	124.64	0.020	20.15
ZnO/EP (650 °C)	−0.428	4.20	240.34	105.51	0.048	7.57
CS–GO–ZnO/EP (uncalcined)	−0.423	4.64	267.26	106.35	0.053	7.12
CS–GO–ZnO/EP (500 °C)	−0.431	6.428	522.28	145.08	0.074	7.71
CS–GO–ZnO/EP (650 °C)	−0.502	27.56	471.14	97.34	0.320	1.21

**Table 5:** Fitted EIS parameters extracted from equivalent electrical circuits for the GO/EP, binary ZnO/EP, ternary hybrid CS–GO–ZnO/EP composite coatings.

Coating Sample	R_s_ (Ω)	R_coat_ (Ω)	CPE_of_ (μΩ^−1^ cm^−2^ s)	R_ct_ (Ω)	CPE_c_ (μΩ^−1^ cm^−2^ s)	X^2^
GO/EP	1.181	1.61 × 10^3^	36.5	298	112	0.028
ZnO/EP (uncalcined)	2.65	3.47 × 10^4^	680	4.36 × 10^3^	62.3	0.11
ZnO/EP (500 °C)	3.13	2.12 × 10^3^	48.5	2.43 × 10^5^	29.9	0.018
ZnO/EP (650 °C)	6.13	2.06 × 10^4^	252.8	4.95 × 10^3^	56.7	0.052
CS–GO–ZnO/EP (uncalcined)	7.03	6.55	60.6	2.97 × 10^3^	0.50	0.07
CS–GO–ZnO/EP (500 °C)	5.08	1.06 × 10^5^	97.3	9.37 × 10^3^	94	0.025
CS–GO–ZnO/EP (650 °C)	2.8	1.65 × 10^3^	0.97	1.24 × 10^3^	117	0.01

## Data Availability

The original contributions presented in this study are included in the article. Further inquiries can be directed to the corresponding authors.

## Abbreviations

The following abbreviations are used in this manuscript:

CS	Chitosan
GO	Graphene oxide
2D	Two-dimensional
ZnO	Zinc oxide
FTIR	Fourier transform infrared spectroscopy
XRD	X-ray diffraction
FE-SEM	Field-emission scanning electron microscopy
EP	Epoxy
AFM	Atomic force microscopy
OCP	Open-circuit potential
EIS	Electrochemical impedance spectroscopy
PDP	Potentiodynamic polarization
E_corr_	Corrosion potential
I_corr_	Corrosion current density
β_a_	Anodic Tafel slope
β_c_	Cathodic Tafel slope
R_p_	Polarization resistance
CR	Corrosion rate
R_s_	Solution resistance
R_ct_	Charge-transfer resistance
R_coat_	Coating resistance
CPE_coat_	Coating constant phase element
CPE_dl_	Double-layer constant phase element
WCA	Water contact angle
